# Interventions for people living with dementia: updates from 2024 Lancet Commission

**DOI:** 10.1002/alz.085068

**Published:** 2025-01-09

**Authors:** Andrew Sommerlad, Jonathan D Huntley, Kathy Y Liu, Sergi Costafreda Gonzalez, Geir Selbaek, Suvarna Alladi, David Ames, Sube Banerjee, Alistair Burns, Carol Brayne, Nick C Fox, Cleusa P Ferri, Laura N. Gitlin, Robert J Howard, Helen C Kales, Mika Kivimaki, Eric B Larson, Noeline Nakasujja, Kenneth Rockwood, Quincy M Samus, Kokoro Shirai, Archana Singh‐Manoux, Lon S. S. Schneider, Sebastian Walsh, Yao Yao, Naaheed Mukadam, Gill Livingston

**Affiliations:** ^1^ University College London, London United Kingdom; ^2^ Division of Psychiatry, University College London, London United Kingdom; ^3^ Camden and Islington NHS Foundation Trust, London United Kingdom; ^4^ Norwegian National Advisory Unit on Aging and Health, Vestfold Hospital Trust, Tønsberg Norway; ^5^ Faculty of Medicine, University of Oslo, Oslo Norway; ^6^ Oslo University Hospital, Oslo Norway; ^7^ National Institute of Mental Health and Neurosciences [NIMHANS], Bengaluru, Karnataka India; ^8^ The University of Melbourne, Melbourne, VIC Australia; ^9^ National Ageing Research Institute, Melbourne, VIC Australia; ^10^ University of Nottingham, Nottingham United Kingdom; ^11^ University of Manchester, Manchester United Kingdom; ^12^ Cambridge Public Health, University of Cambridge, Cambridge United Kingdom; ^13^ Dementia Research Centre, UCL Queen Square Institute of Neurology, London United Kingdom; ^14^ Hospital Alemão Oswaldo Cruz, São Paulo, São Paulo Brazil; ^15^ Universidade Federal de São Paulo (UNIFESP), São Paulo, São Paulo/SP Brazil; ^16^ Drexel University, Philadelphia, PA USA; ^17^ Division of Psychiatry, University College London, London, London United Kingdom; ^18^ University of California, Davis, Sacramento, CA USA; ^19^ Kaiser Permanente Washington Health Research Institute, Seattle, WA USA; ^20^ Department of Medicine, University of Washington, Seattle, WA USA; ^21^ College of Health Sciences, Makerere University, Kampala Uganda; ^22^ Dalhousie University, Halifax, NS Canada; ^23^ Johns Hopkins University, Baltimore, MD USA; ^24^ Osaka University, Osaka Japan; ^25^ Université de Paris, Inserm U1153, Epidemiology of Ageing and Neurodegenerative Diseases, Paris France; ^26^ Department of Psychiatry and Behavioral Sciences, Keck School of Medicine, University of Southern California, Los Angeles, CA USA; ^27^ Cambridge Public Health, Cambridge United Kingdom; ^28^ Peking University, Beijing China

## Abstract

**Background:**

The progressive nature of dementia and the complex needs means that people living with dementia require tailored approaches to address their changing care needs over time. These include physical multimorbidity, psychological, behavioural, and cognitive symptoms and possible risks arising from these and helping family caregivers. However, provision of these interventions is highly variable between and within countries, partly due to uncertainty about their efficacy and scarce resources. In the 2024 update of the Lancet Commission we aimed to summarise published evidence about the effect of non‐pharmacological interventions for people with dementia and their carers on cognition, neuropsychiatric symptoms and other person‐centred outcomes.

**Method:**

We reviewed and summarised evidence according to expert consensus opinion.

**Result:**

There is moderate‐quality evidence from a Cochrane review of 25 studies for effect of cognitive stimulation therapy on cognition; 1.99 (1.24‐2.74) Mini‐Mental State Examination points higher compared to control groups, and clinically relevant improvements in communication and social interaction. Multicomponent interventions for family carers reduce family carer depression, burden, or stress and are cost‐effective but remote delivery of these interventions was not better than care as usual. A meta‐analysis of 7 studies of tailored activity programmes for people with dementia found a moderate effect on improving quality of life (standardised ES Cohen’s d 0.79, 0.39–1.18; 7 studies, n = 160), decreasing neuropsychiatric symptoms (0.62; 0.40–0.83) and decreasing carer burden (0.68, 0.29–1.07) but there is little evidence on cost‐effectiveness. Exercise interventions were not effective in improving neuropsychiatric symptoms, cognition or functioning. We discuss evidence for other treatments for specific neuropsychiatric symptoms.

**Conclusion:**

There is developing evidence for benefit of psychological and social interventions on key outcomes including cognition, neuropsychiatric symptoms and quality of life, with sufficient strength of evidence and cost‐effectiveness to justify these being implemented and offered routinely to people with dementia. Interventions generally should be tailored to specific symptoms and individualised to patient preferences and goals. Most interventions have been tested in majority ethnic populations in high income countries: future interventions should be co‐designed with local communities to ensure that they are appropriate for the context, culture, beliefs and practices.

